# Two Cases of Trocar Bending of the Injector During iStent Inject W Implantation

**DOI:** 10.7759/cureus.107218

**Published:** 2026-04-17

**Authors:** Masaki Tanito, Hinako Ohtani, Chisako Ida, Keigo Takagi, Yuto Yoshida, Kazunobu Sugihara, Sachiko Kaidzu

**Affiliations:** 1 Department of Ophthalmology, Shimane University Faculty of Medicine, Izumo, JPN

**Keywords:** device complication, istent inject w, minimally invasive glaucoma surgery (migs), surgical complication, trabecular micro-bypass, trocar bending

## Abstract

We report two cases of trocar tip bending during implantation of the iStent inject W in combination with cataract surgery. In both cases, the trocar within the insertion tube was bent at a similar location and direction. In Case 1, the bending was recognized after stent deployment, although surgical video review confirmed that it had already been present before deployment. In Case 2, the bending was identified prior to insertion of the injector tip into the anterior chamber. In both cases, the stents initially failed to be properly implanted, and successful implantation was achieved using a new injector. Postoperative courses were uneventful except for transient hyphema. Stereomicroscopic examination revealed that the trocar was bent approximately 0.9-1.0 mm from the tip. These findings suggest that bent-type trocar damage can occur when the exposed trocar tip catches on the corneal stroma during passage through the corneal incision, particularly with the iS2 injector system. Recognition of this damage before stent deployment and prompt replacement of the device are essential to avoid improper implantation and ensure surgical safety.

## Introduction

A group of procedures collectively referred to as Minimally Invasive Glaucoma Surgery (MIGS) are primarily performed in combination with cataract surgery, aiming to achieve visual rehabilitation and a modest reduction in intraocular pressure (IOP) in patients with glaucoma associated with cataracts. Surgery using the iStent trabecular micro-bypass system is one of the representative MIGS procedures. Implantation of the second-generation iStent inject (Glaukos Corporation, San Clemente, CA) in combination with cataract surgery is associated with a significantly greater postoperative reduction in IOP compared with cataract surgery alone in eyes with primary open-angle glaucoma (POAG), while maintaining a low rate of vision-threatening postoperative complications [1]. Currently, a modified second-generation device, iStent inject W, which features a wider stent flange than the iStent inject, is available for clinical use. The IOP-lowering effect of the iStent inject W has been reported to be comparable to or greater than that of the first-generation iStent [2]. In addition, faster postoperative visual recovery than with microhook trabeculotomy [3] and minimal induction of higher-order aberrations following surgery [4] have been reported.

The stents of the iStent inject are mounted in a skewered fashion on a trocar within the insertion tube of a dedicated injector system, and the trocar has a flared tip (trocar splay). During surgery, with the trocar inserted into the trabecular meshwork, pressing the injector button deploys the stent, which is then implanted into the trabecular meshwork/Schlemm's canal. As an intraoperative complication of iStent surgery, stent malposition may occur. In addition, trocar damage has been recognized as another complication [5,6]. Among these, trocar splay caused by tensile failure during stent deployment has been reported as the most typical fracture pattern [5,6]. Here, we report two cases of a distinct type of trocar damage, different from tensile failure, that occurred during iStent inject W surgery.

## Case presentation

Case 1

A 67-year-old Japanese woman had been receiving topical treatment for POAG in both eyes (OU) for 10 years. She was referred to Shimane University Hospital due to bilateral cataracts and progressive visual field loss in the left eye (OS). At the initial visit, best-corrected visual acuity (BCVA) was 1.2 with −4.5 D myopic correction in the right eye (OD) and 0.7 with −6.0 D myopic correction OS. IOP, measured by Goldmann applanation tonometry (GAT), was 15 mmHg OD and 17 mmHg OS under treatment with latanoprost once daily OU and a fixed combination of timolol/dorzolamide twice daily OS. The cornea was clear OU. The anterior chamber angle was wide open with trace pigmentation and no peripheral anterior synechiae (PAS) OU. Nuclear cataracts were present OU without laterality. The cup-to-disc ratio (vertical × horizontal) was 0.9 × 0.8 OU, and retinal nerve fiber layer defects (NFLD) were observed OU.

Optical coherence tomography (OCT) using the RS-3000 Advance 2 (Nidek, Gamagori, Japan) demonstrated thinning of the papillomacular bundle OS. The mean deviation (MD) on the Humphrey 30-2 visual field test (Humphrey Field Analyzer, Carl Zeiss Meditec, Dublin, CA, USA) was −1.46 dB OD and −21.19 dB OS, showing visual field defects consistent with the fundus findings OU. Corneal endothelial cell density (CECD) was 2,380 cells/mm² OD and 2,121 cells/mm² OS, and central corneal thickness (CCT) was 487 μm OD and 450 μm OS, measured by specular microscopy (EM-3000, Tomey, Nagoya, Japan). Anterior chamber flare was 6.9 pc/ms OD and 6.2 pc/ms OS, measured using a laser flare meter (FM-600α, Kowa, Tokyo, Japan).

**Figure 1:**
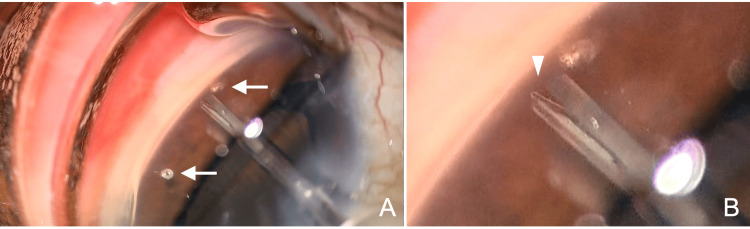
Intraoperative findings in Case 1 (right eye). (A) Two stents (arrows) that fail to be inserted into the trabecular meshwork and are dislodged into the anterior chamber. The injector tip is inserted into the eye through a 2.2-mm corneal incision. (B) Under the surgical microscope, the trocar within the insertion tube appears to be bent (arrowhead).

Combined cataract surgery with implantation of the iStent inject W was performed OD (Video 1). Phacoemulsification was performed through a 2.2-mm temporal corneal incision, and an intraocular lens (+17.0 D, XY-1EM, HOYA, Tokyo, Japan) was implanted in the capsular bag. Subsequently, under gonioscopic visualization using a Swan-Jacob Gonio Prism (Ocular Instruments, Bellevue, WA, USA), stent implantation was attempted using the injector. The distal tip of the insertion tube was gently pressed against the trabecular meshwork, and the stents were deployed; however, neither of the two stents was successfully implanted into the trabecular meshwork (Figure 1A). Intraoperative microscopic observation suggested bending of the trocar within the insertion tube (Figure 1B). The surgeon did not recognize the trocar bending prior to stent deployment. The improperly deployed stents were removed from the eye using anterior capsulorhexis forceps (Ikeda micro capsulorhexis forceps, Inami, Tokyo, Japan). A new injector was then used to successfully implant two stents in the nasal angle, and the surgery was completed. Postoperatively, 1.5% levofloxacin and 0.1% betamethasone phosphate eye drops were administered four times daily for three weeks. No significant postoperative complications were observed, except for mild hyphema (grade 100 according to the Shimane University hyphema grading system [7]) on postoperative day 1.

**Video 1:** Surgical findings of Case 1.

**Figure 2:**
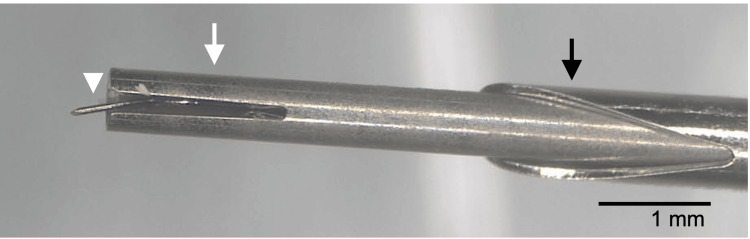
Stereomicroscopic observation of the injector tip used in Case 1. In the iS2 injector system, when the injector tip is inserted into the eye, the introducer tip (black arrow) is pushed down by the peripheral corneal tissue, resulting in exposure of the insertion tube (white arrow). The trocar (arrowhead) within the insertion tube is observed to be bent at approximately 0.9 mm from the tip. This image is provided by Glaukos (Aliso Viejo, CA, USA).

Postoperative examination of the injector tip using a stereomicroscope revealed that the trocar within the insertion tube was bent at approximately 0.9 mm from the tip (Figure 2). At four months postoperatively, BCVA OD was 1.0, and IOP OD was 14 mmHg under treatment with a fixed combination of timolol/dorzolamide twice daily. CECD was 2,179 cells/mm², CCT was 479 μm, and anterior chamber flare was 8.6 pc/ms. The Humphrey visual field MD was −1.90 dB OD.

Case 2

A 78-year-old Japanese man had been receiving topical treatment for POAG OU for 14 years. He was referred to Shimane University Hospital due to decreased visual acuity caused by cataract OS. At the initial visit, BCVA was 1.0 with −3.25 D myopic correction OD and 0.7 with −8.25 D myopic correction OS. IOP (GAT) was 14 mmHg OU under treatment with a fixed combination of latanoprost/timolol once daily OU and a fixed combination of brimonidine/dorzolamide twice daily OU. The cornea was clear OU. The anterior chamber angle was wide open with trace pigmentation and no PAS OU. Nuclear cataracts were present in both eyes, and a posterior subcapsular cataract was noted OS. The cup-to-disc ratio was 0.9 × 0.8 OD and 1.0 × 0.8 OS, and NFLD was observed OU. OCT (RS-3000 Advance 2) demonstrated that the papillomacular bundle was preserved OU. MD on the Humphrey 30-2 visual field test (Carl Zeiss Meditec) was −7.99 dB OD and −4.50 dB OS, showing visual field defects consistent with the fundus findings OU. CECD was 2,769 cells/mm² OD and 2,732 cells/mm² OS, and CCT was 533 μm OD and 526 μm OS, measured by specular microscopy (EM-3000). Anterior chamber flare was 14.1 pc/ms OD and 13.0 pc/ms OS, measured using a laser flare meter (FM-600α).

**Figure 3:**
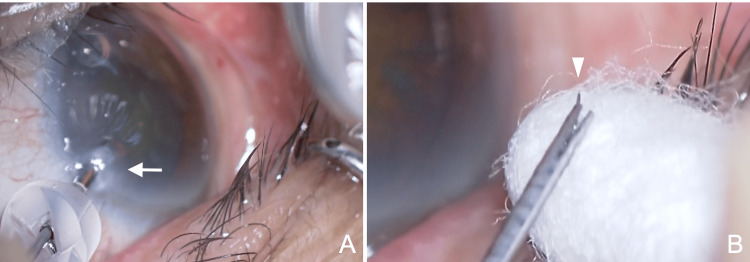
Intraoperative findings in Case 2 (left eye). (A) The injector tip is inserted into the eye through a corneal side port created with a 20G MVR knife. The injector tip catches on the corneal tissue, and stress on the cornea is observed (arrow). (B) Under the surgical microscope, bending of the trocar within the insertion tube is observed (arrowhead).

Combined cataract surgery with implantation of the iStent inject W was performed in the left eye (Video 2). A corneal side port was created superotemporally using a 20G MVR lance (1.2 mm, Mani, Utsunomiya, Japan). The anterior chamber was filled with two types of ophthalmic viscosurgical devices: Shellgan (Santen, Osaka, Japan) and ProVisc (Alcon Japan, Tokyo, Japan). Subsequently, under gonioscopic visualization using a Swan-Jacob Gonio Prism (Ocular Instruments), stent implantation was attempted using the injector. During insertion, the distal tip of the insertion tube caught on the corneal side port and could not be advanced into the anterior chamber (Figure 3A). Intraoperative microscopic observation of the injector tip revealed bending of the trocar within the insertion tube (Figure 3B). A new injector was then used to successfully implant two stents in the nasal angle. Subsequently, cataract surgery was performed through a 2.2-mm nasal corneal incision, and an intraocular lens (+18.0 D, XY-1EM, HOYA) was implanted in the capsular bag, completing the procedure. Postoperatively, 1.5% levofloxacin and 0.1% betamethasone phosphate eye drops were administered four times daily for three weeks. No significant postoperative complications were observed, except for hyphema (300 according to the Shimane University hyphema grading system [7]) on postoperative day 1.

**Video 2:** Surgical findings of Case 2.

**Figure 4:**
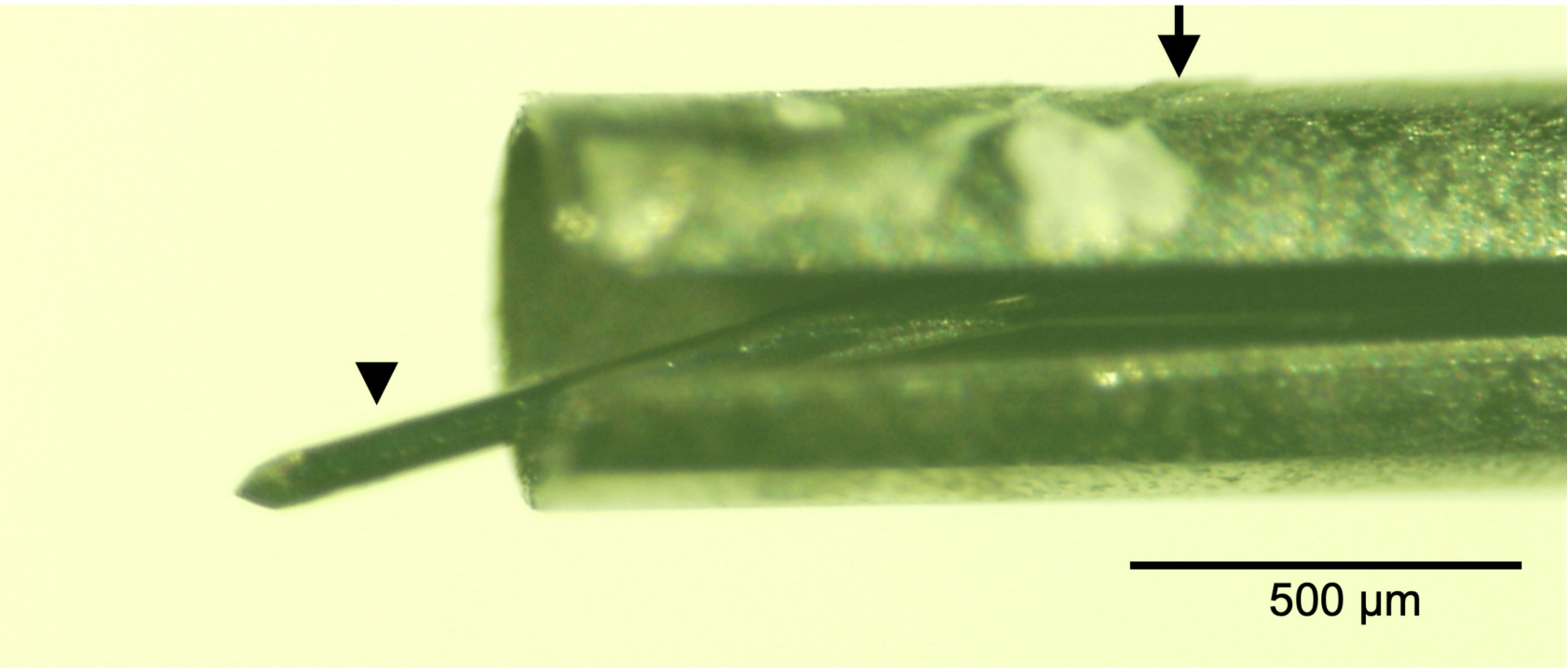
Stereomicroscopic observation of the injector tip used in Case 2. The trocar (arrowhead) within the insertion tube (arrow) is observed to be bent at approximately 1 mm from the tip.

Postoperative stereomicroscopic examination of the injector tip revealed that the trocar within the insertion tube was bent at approximately 1 mm from the tip (Figure 4). The direction of the trocar bending was the same as in Case 1. At two months postoperatively, BCVA OS was 1.0, and IOP OS was 11 mmHg under treatment with a fixed combination of brimonidine/dorzolamide twice daily. CECD was 2,881 cells/mm² OS, CCT was 522 μm OS, and anterior chamber flare was 20.1 pc/ms OS.

## Discussion

In Case 1, the surgeon recognized the trocar bending after stent deployment. However, subsequent review of the surgical video revealed that the trocar had already been bent prior to deployment. In Case 2, the trocar bending was identified before insertion of the trocar tip into the anterior chamber. In both cases, the location and direction of the bending were highly similar (Figures 2, 4). These findings suggest that the trocar bending in both cases likely occurred when the trocar tip caught on the corneal stromal tissue during attempted insertion into the anterior chamber.

An analysis of 808 surgical complication reports of various iStent models from the manufacturer and user facility device experience (MAUDE) database showed that device malposition was reported as an intraoperative complication across all models, including the first-generation iStent, iStent inject, iStent inject W, and iStent infinite, whereas trocar damage was reported only for the iStent inject W and iStent Infinite models [5]. Among reports specifying damage type, tensile failure was the most common (19 of 29 cases, 65.5%), followed by bending (7 of 29 cases, 24.1%) and shear damage (3 of 29 cases, 10.3%) [5]. In addition, the most typical mechanism of tensile failure is considered to be trocar bias when pressing the trocar tip against the trabecular meshwork, with the resulting misalignment leading to splay collision [6]. Previously reported types of trocar damage have generally been attributed to intraocular manipulation [5]. However, the two cases presented here indicate that bent-type trocar damage can also occur due to mechanical catching of the trocar tip on tissue during passage through the corneal incision.

Recently, the injector design of the iStent inject W has been modified and introduced into clinical practice (since 2025 in Japan). With this modification, the limitation on the number of stent deployments has been removed, and the rigidity of the insertion tube has been increased. In the conventional injector, the trocar tip was exposed by the surgeon after insertion of the insertion tube into the eye. In contrast, the new injector (iS2) is equipped with an introducer tip surrounding the insertion tube. In the iS2 system, as the insertion tube passes through the corneal incision, the introducer tip is pushed back by the corneal tissue, resulting in automatic exposure of the trocar tip. Therefore, in the iS2, the trocar tip may already be exposed while the insertion tube is still traversing the corneal incision. Both cases in this report involved the iS2-type device. In these cases, it is highly likely that the already exposed trocar tip caught on the corneal stroma within the incision, resulting in bent-type trocar damage. Furthermore, the angled configuration between the insertion tube and the injector body in the iS2 may have contributed to this event, particularly for surgeons accustomed to the conventional straight-type injector.

The type of trocar damage observed in this report occurs during the process of approaching the trabecular meshwork. Therefore, recognizing the damage before stent deployment is considered the most appropriate management. It is important for surgeons to be aware of this specific pattern of trocar damage. In this regard, reporting these two cases is meaningful for improving surgical safety. For Case 1, the event has been reported to the iStent manufacturer. At present, there is no consensus as to whether the cases described in this manuscript are attributable to the technique of a specific surgeon or to issues related to the injector design. If similar cases are reported by other institutions or surgeons, more generalized countermeasures may need to be considered.

## Conclusions

We experienced two cases of trocar tip bending during iStent inject W surgery. Both cases involved the use of iS2 injectors and were characterized by trocar bending at approximately 1 mm from the tip, resistance during insertion, and successful reimplantation using a new device. This was considered to be a bent-type trocar damage, likely caused by the trocar tip catching on the corneal stroma during insertion of the insertion tube into the anterior chamber. When this type of trocar damage is recognized, stent deployment should be avoided, and a new device should be used. Awareness of this type of trocar damage is important to prevent unnecessary stent deployment.
